# Mixture Effects of Bisphenol A and Its Structural Analogs on Estrogen Receptor Transcriptional Activation

**DOI:** 10.3390/toxics11120986

**Published:** 2023-12-04

**Authors:** Handule Lee, Juyoung Park, Kwangsik Park

**Affiliations:** College of Pharmacy, Dongduk Women’s University, Seoul 02748, Republic of Korea

**Keywords:** endocrine disruption, mixture toxicity, estrogen receptor, bisphenol A, BPA analogs

## Abstract

Bisphenol A (BPA) exposure has been widely linked to endocrine-disrupting effects. Recently, many substitutes for BPA have been developed as safe structural analogs. However, they have still been reported to have similar adverse effects. The current study evaluated the effects of bisphenol A and eight structural analogs on the transcription of estrogen receptor alpha (ERα). The effects of binary and ternary mixtures prepared from different combinations of BPA analogs were also evaluated for transcription activity. The measured data of the mixtures were compared to the predicted data obtained by the full logistic model, and the model deviation ratio (MDR) was calculated to determine whether the effects were synergistic, antagonistic, or additive. Overall, the results suggest that the effect of bisphenol compound are additive in binary and ternary mixtures.

## 1. Introduction

Bisphenol A [BPA, 4′4-(Propane-2,2-diyl)diphenol] is a crystalline solid substance used in the production of epoxy resins and polycarbonate plastics, which are found in many consumer and industrial products. Generally, humans are exposed to BPA through food packaging materials, water bottles, and lacquer coatings for cans. Furthermore, occupational workers are also exposed through direct contact with skin or inhalation. Due to its increased production and use worldwide (several million tons every year), it is now ubiquitously found in the environment including air, water, soil, house dust, and foodstuffs, as well as in human samples such as urine, blood, amniotic fluid, saliva, and breast milk [1,2,3]. BPA exposure has been widely linked to adverse health effects; it is known to be associated with the incidence of growth disruption, halting normal development, infertility, endocrine system disruption, immune system suppression, and carcinogenicity in animal models and epidemiological human studies [4,5,6]. BPA interacts with several biological receptors such as estrogen receptor (ER), androgen receptor (AR), and thyroid hormone receptor (THR), resulting in endocrine-disrupting effects. These effects lead to adverse outcomes such as reproductive and developmental issues in the offspring, reproductive dysfunction, neurotoxicity, mutagenesis, and even cancer development [7,8,9,10,11].

Due to the adverse health effects caused by BPA, the European Union (EU) and many countries including the United States (US), Canada, China, Japan, and Korea have successively enacted laws and regulations to restrict or prohibit the use of BPA. The US Food and Drug Administration (FDA) banned its use in food packaging in 2010; the EU banned its use in baby bottles in 2011; the European Food Safety Authority (EFSA) established a tolerable daily intake of 50 μg/kg/day in 2015; the European Chemicals Agency (ECHA) recognized it as an endocrine disruptor in humans in 2017. However, despite the regulations, BPA is still used in various fields including healthcare [5,12].

In recent years, the BPA structural analogs such as Bisphenol AF (BPAF), Bisphenol B (BPB), Bisphenol F (BPF), Bisphenol S (BPS), and Bisphenol Z (BPZ) have increasingly been adopted due to the hazard of BPA [13]. However, the reports on the endocrine toxicity of these analogs are increasing [14,15,16]. BPS, although it has been introduced as a safe, is known to have similar endocrine toxicity to BPA. Interactions between BPS and estrogen receptor alpha (ERα), subsequent disruption of the reproductive neuroendocrine system and immunotoxic potential via receptor binding have been evaluated in several studies. BPS was also found to alter the secretion of progesterone and estradiol in cultured ovine granulosa cells, which disrupts steroidogenesis [17,18]. Kitamura et al. reported the endocrine-disrupting activity of BPA and 19 related compounds. In the reports, several BPA analogs exhibited estrogenic activity in the human breast cancer cell line MCF-7 [19]. Testicular toxicity of BPS, BPAF, and tetrabromobisphenol A (TBBPA) was also investigated in mouse spermatogonial cells, and they exhibited higher toxicities including dose- and time-dependent alterations in nuclear morphology and cell cycle, DNA damage responses, and perturbation of the cytoskeleton [20]. To evaluate the toxicity potential of BPA substitutes, a toxicokinetic study was also performed after oral and intravenous administration in pigs [21]. Bioavailability and the internal dose of the substitutes are well-known key factors for the evaluation of human exposure risk. A test using *Diaphanosomacelebensis*, BPA, BPS, and BPF showed transcriptional modulation of genes which is related to the ecdysteroid pathway, indicating that these compounds can disrupt the normal endocrine system function of *D. celebensis* [22].

In human biomonitoring studies of BPA exposure, BPA and its substitutes were measured in the same blood and/or urine samples. This suggested that the co-deposition in target organs may increase the risk of bisphenol compounds [23,24]. It is therefore necessary to evaluate mixtures of bisphenol compounds to obtain a more realistic and reliable assessment of health risks compared to assessing individual chemicals [25]. Even with the increasing reports on the exposure of a mixture of bisphenol compounds, only a few of them have been published on the estrogenic activity of the mixtures [26,27]. Some tests on the mixture effects of BPA were performed with non-bisphenol compounds such as heavy metals, phthalates, and alkylphenols [28,29,30].

In this study, BPA and its eight analogs were selected and screened for estrogen receptor alpha (ERα) transactivation activity (agonistic/antagonistic) based on the OECD Test guideline 455 using HeLa 9903 cells [31]. Some of the compounds were found to be agonists, some were antagonists, and some showed no effects. After the evaluation of the single compounds, the transcriptional activity of binary and ternary mixtures (with combinations of agonist/agonist and antagonist/antagonist) was evaluated. In addition, calculated prediction values of the mixtures using the full logistic model (FLM) were also obtained and compared with the measured values. This study aimed to provide a concept for the prediction of endocrine-disrupting effects of bisphenol compounds that are simultaneously exposed to humans. The results of this study will help to develop and implement regulations for numerous BPA compound mixtures found in consumer products with potential endocrine-disrupting properties.

## 2. Materials and Methods

### 2.1. Test Chemicals

Test chemicals of BPA and its analogs were purchased from Sigma-Aldrich (St. Louis, MO, USA) and Tokyo Chemical Industry (TCI, Tokyo, Japan). They were first dissolved in 90% Dimethyl sulfoxide (DMSO) and then serially diluted to prepare test concentrations. The final concentration of the DMSO in media was 0.1%. The chemical name, abbreviation, structure, CAS number, test concentrations, and vendors are shown in Table 1. The reference chemicals for ERα transactivation assay, 17α-estradiol, and tamoxifen, were purchased from Sigma-Aldrich, and flutamide, 17β-estradiol (E2), corticosterone, and 17α-methyltestosterone were purchased from Tokyo Chemical Industry.

### 2.2. Cell Culture

The hERα-HeLa-9903 (HeLa 9903) cell line (JCRB1318), which expresses human estrogen receptor α (hERα), was obtained from the Japanese Collection of Research Bioresources (JCRB) cell bank (Osaka, Japan), and cultured in phenol red-free Eagle’s minimum essential medium (EMEM) supplemented with 10% dextran-coated charcoal-treated fetal bovine serum (DCC-FBS) and kanamycin (60 mg/L). Plastic wares with no estrogenic activity were used in this study according to OECD TG 455 [31].

### 2.3. Cytotoxicity Test

To achieve the appropriate test chemicals concentration for transactivation assay, the cytotoxicity tests were performed using 3-(4,5-Dimethylthiazol-2-yl)-2,5-Diphenyltetrazolium Bromide (MTT) assay. After 24 h stabilization in 96-well plates, HeLa 9903 cells were treated with test chemicals at wide ranges of concentrations (10^−11^ M~10^−3^ M) for 24 h. After exposure, 100 μL of the MTT solution (5 mg/mL) was added, and the cells were incubated for 2 h at 37 °C. The cells were treated with 100 μL of DMSO to solubilize the purple formazan, and the absorbance was quantified at 570 nm using the microplate spectrophotometer system (Spark^®^, TECAN, Männedorf, Switzerland). The concentration at which cell viability began to decrease was selected as the highest concentration for the ERα transactivation assay.

### 2.4. Preparation of Mixtures

Binary mixtures were prepared by combining two bisphenol compounds; agonist/agonist, and antagonist/antagonist. For the test concentrations of binary mixtures, the highest test concentration (master solution) was achieved by mixing individual chemicals where the final concentration was designed to be 100-fold EC_50_ of the individual compound for the agonist, and 100-fold IC_50_ of the individual compound for the antagonist (equi-effect basis). EC_50_ and IC_50_ are shown in Table 2. The assay was designed to obtain full concentration–response curve. The master solution of each binary mixture was then serially diluted for testing. Ternary mixtures were prepared by combining three bisphenol compounds—agonist/agonist/agonist, and antagonist/antagonist/antagonist—where the final concentration was designed to be 100-fold EC_50_ of the individual compound for the agonist, and 100-fold IC_50_ of the individual compound for the antagonist (equi-effect basis). In the case of BPB, the EC_50_ was too high to prepare 100-fold mixtures due to cytotoxicity at the master solution. So, the mixture containing BPB was designed to be 0.05-fold in binary mixture, and 0.25-fold in ternary mixture, as described in Table 3. Information on the master solution of the binary and ternary mixtures is presented in Table 3. No cytotoxicity was observed at the master solution.

### 2.5. Stably Transfected Transactivation (STTA) Assay for Estrogen Receptor Alpha

For the estrogen receptor agonist and antagonist tests, the ERa transactivation assay was performed based on the OECD TG 455 using cultured HeLa 9903 cells, which stably express the ERα gene containing a firefly luciferase gene as the reporter gene [31]. After subculturing the cells to a density of 75~90% in a 100 mm culture dish, the cells were transferred to a 96-well plate at 1 × 10^4^ cells/100 μL/well, stabilized for 3 h, and then treated with the test chemicals for 24 h. After exposure, the luciferase activity was measured using a commercial luciferase assay reagent (E2510, Promega, Maidison, WI, USA). For the ERα transcription agonist assay, the normalized mean value of the luminescence signal (Luciferase activity) of the test wells was divided by that of the positive chemical (1 nM 17β-estradiol) to obtain relative transcriptional activity. The concentration–response curves of the test chemicals were processed to calculate the PC_10_ and PC_50_ values, which show 10% and 50% of the transcriptional activity of positive chemicals, respectively, using software provided by the OECD Test guideline. 17β-estradiol, 17α-estradiol, 17α-methyltestosterone, and corticosterone were used as the reference chemicals for the ERα transactivation assay to monitor the stability of the assay system. For the antagonist assay, test wells were spiked with 25 pM 17β-estradiol. Tamoxifen and flutamide were used as reference chemicals. The normalized mean value of the luminescence signal (Luciferase activity) of the test wells was divided by that of spiked 25 pM 17β-estradiol (100%) to obtain relative transcriptional activity. RPC_Max_ for the agonist and IC_30_ values for the antagonist were used for the positive and negative decision criteria, according to the OECD TG 455. The calculations of PC_10_, PC_50,_ and RPC_Max_ in ERα agonist assay and IC_30_ and IC_50_ in ERα antagonist assay can be made by using the spreadsheet available together with the Test Guideline on the OECD public website [32,33].

### 2.6. The Prediction of ERα Transactivation of the Mixtures by the Full Logistic Model

The mixture’s ERα transactivation assay was performed as described above and the measured data were obtained. Estimation of ERα transactivation by the chemical mixtures was performed with the full logistic model (FLM) with GraphPad Prism (V.9.3, Dan Diego, CA, USA) [34,35,36]. FLM used four parameters including Hill slope, the maximum and minimum response values, and EC_50_ of individual chemicals. The equation for FLM was as follows:

$$E_{mix}=\left[MAX_{1}+\frac{MIN_{1}-MAX_{1}}{1+{\left(\frac{C_{1}}{EC_{50,1}}+\frac{C_{2}}{EC_{50,2}}\right)}^{P_{1}}}\right]\cdot \left[\frac{\frac{C_{1}}{EC_{50,1}}}{\left(\frac{C_{1}}{EC_{50,1}}+\frac{C_{2}}{EC_{50,2}}\right)}\right]+\left[MAX_{2}+\frac{MIN_{2}-MAX_{2}}{1+{\left(\frac{C_{2}}{EC_{50,2}}+\frac{C_{1}}{EC_{50,1}}\right)}^{P_{2}}}\right]\cdot \left[\frac{\frac{C_{2}}{EC_{50,2}}}{\left(\frac{C_{2}}{EC_{50,2}}+\frac{C_{1}}{EC_{50,1}}\right)}\right]$$

*MAX*: the maximal value of the dose–response curve in the test of individual chemicals;
*MIN*: the minimal value of the dose–response curve in the test of individual chemicals;
*EC*_50_: the half value of maximal effective concentration;
*P*: hill slope;
*C*: the concentration of individual chemicals in the test mixture;
The number indicates individual chemicals in the test mixture.

The model for the prediction of the mixture effect is based on the concentration–response curves of each chemical component obtained by the ERα transactivation assay. The parameters of the concentration–response curves for the individual chemicals were calculated using GraphPad Prism, as shown in Table 2. The parameter values were substituted into the FLM formulas. The measured values were compared to the predicted values obtained by the FLM.

### 2.7. The Assessment of Model Deviation Ratio (MDR)

To verify the difference between the measured and the predicted results, the Model Deviation Ratio (MDR) approach was applied. MDR is defined as the ratio obtained by dividing the expected value by the measured value at respective concentrations [37,38]. In this study, the ratio between the predicted effects calculated by FLM and the measured values at seven concentration points was obtained. MDR values lower than 0.50 are indicators of the synergistic behavior of compounds present in a mixture while values over 2.00 justify the statement of antagonistic action. MDR values within 0.50~2.00 indicate additive activity.

## 3. Results

### 3.1. Cell Viability and Test Concentration

**Figure 1:**
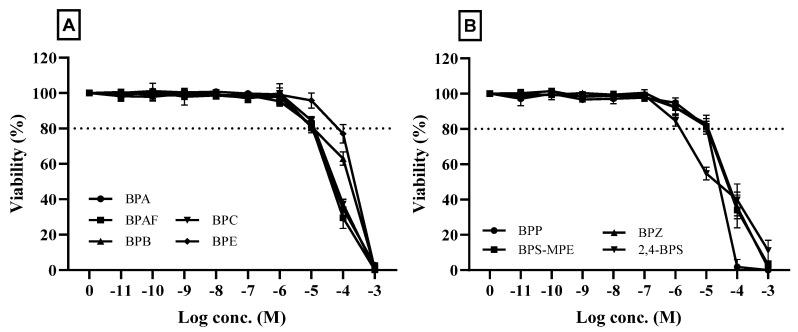
Effects of test chemicals on the viability of cultured HeLa 9903 cells. Cell viability was assessed by MTT assays, and the results are presented as a percentage of the control group viability. Cells were treated with indicated concentrations for 24 h. The results represent the means of three separate experiments, and the bars represent the standard error. The dotted line in the figure represents a viability of 80%. The figure shows the cytotoxicity of the chemicals that showed positive results in the agonistic or antagonistic tests of estrogen receptor transactivation (*n* = 3). (**A**): Bisphenol A (BPA), Bisphenol AF (BPAF), Bisphenol B (BPB), Bisphenol C (BPC), and Bisphenol E (BPE); (**B**): Bisphenol P (BPP), Bisphenol S(BPS)-MPE, 2,4-dihydroydiphenyl sulfone (2,4-BPS), and Bisphenol Z (BPZ).

To determine the appropriate test concentrations of the test chemicals, a cytotoxicity test was performed using the MTT assay, and the results are shown in Figure 1. The highest test concentration for transactivation assay, which was the concentration that showed the initial decrease in viability, was chosen from the cytotoxicity data, and most chemicals showed a decrease in viability at a concentration of 10^−5^~10^−4^ M. The dotted lines in the figure indicate 80% viability compared with the media control group, which is the baseline for the determination of the highest concentration for the ERα transactivation assay. The concentration which showed a viability of less than 80% was not applied to the assay.

### 3.2. Effects of Bisphenol A and Its Analogs on ERα Transactivation: Agonist Assay

**Figure 2:**
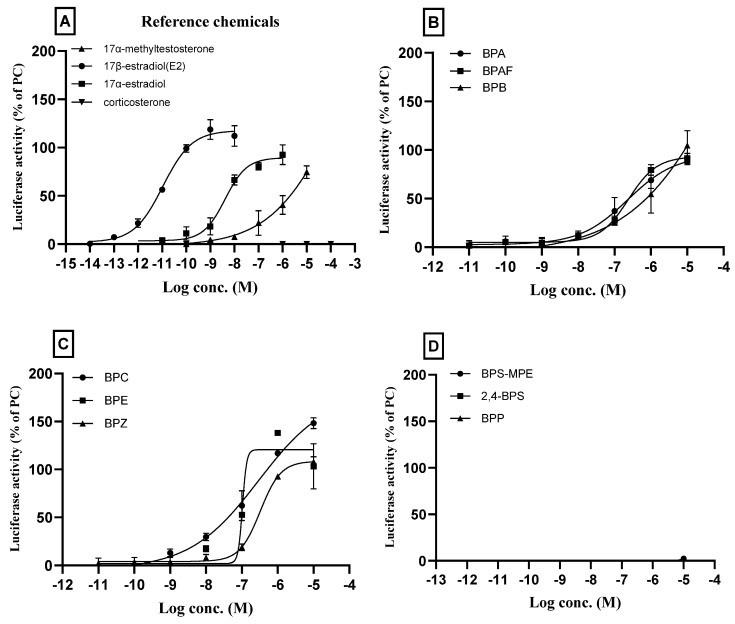
Agonistic assay of bisphenol compounds for estrogenic receptor alpha (ERα) transcriptional activity in cultured HeLa 9903 cell line. The cells were incubated with different concentrations of the respective bisphenol compounds based on the results of the cytotoxicity test, and a transactivation assay was performed. For the ERα transcription agonist assay, the normalized mean values of the luminescence signal (Luciferase activity) of the test wells were divided by that of the positive chemical (1 nM 17β-estradiol) to obtain the relative transcriptional activity. (**A**): The results of reference chemicals; 17β-estradiol, 17α-estradiol, and 17α-methyltestosterone induced ERα transactivation, while corticosterone showed no activity. (**B**): BPA, BPAF, BPB; (**C**): BPC, BPE, BPZ; (**D**): BPS-MPE, 2,4-BPS, BPP. Three separate experiments were performed in the transactivation assay and data were represented as mean ± standard error.

The estrogenic activities of BPA and its eight analogs were evaluated based on OECD TG 455. For the agonist assay of ERα transactivation in cultured HeLa 9903 cells, the reference chemicals 17β-estradiol, 17α-estradiol, and 17α-methyltestosterone and corticosterone were first tested. As shown in Figure 2A, concentration–response transactivation was induced by 17β-estradiol, 17α-estradiol, and 17α-methyltestosterone, with different potencies and efficacies. The results for the reference chemicals were very similar to those provided in the test guideline. When the cultured cells were treated with corticosterone, no sign of transactivation was observed in all the test concentrations, 10^−10^~10^−4^ M, as shown in TG 455. These results confirmed the validity of the performance criteria for the agonist assay in this study.

BPA and its analogs BPAF, BPB, BPC, BPE, and BPZ showed ERα transactivation agonistic activities toward ERα transcription. The concentration–response curves of the chemicals with agonistic effects are shown in Figure 2B,C. BPS-MPE, 2,4-BPS, and BPP did not exhibit agonistic activity (Figure 2D). RPC_Max_, PC_10,_ and PC_50_ of individual chemicals which showed ERα agonistic effects were obtained from the concentration–response curve by using the spreadsheet provided by the OECD TG 455 and are presented in Table 4.

### 3.3. Effects of Bisphenol A and Its Analogs on ERα Transactivation: Antagnoist Assay

**Figure 3:**
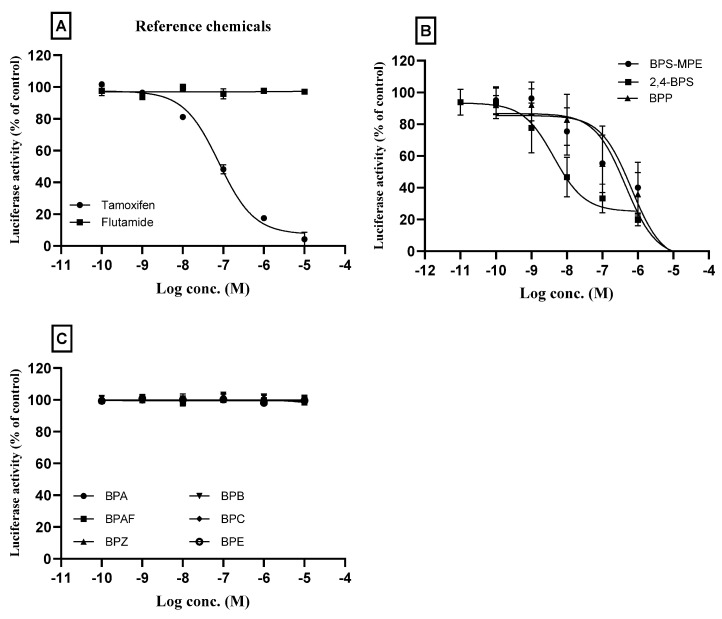
Antagonistic assay of bisphenol compounds for estrogenic receptor alpha (ERα) transcriptional activity in cultured HeLa 9903 cell line. The cells were incubated with different concentrations of bisphenol compounds based on the results of the cytotoxicity test, and an antagonistic assay was performed. For the antagonist assay, test wells were spiked with 25 pM 17β-estradiol. The normalized mean values of the luminescence signal (Luciferase activity) of the test wells were divided by that of the spiked-in control (100%) to obtain the relative transcriptional activity. (**A**): Tamoxifen (positive) and flutamide (negative) were used as the reference chemicals. (**B**): BPS-MPE, 2,4-BPS, and BPP showed positive antagonistic activity to the ERα transactivation. (**C**): BPA, BPAF, BPB, BPC, BPE, and BPZ did not show antagonistic activity to the 17β-estradiol-activated ERα transcription. Three separate experiments were performed in transactivation assay, and data were represented as mean ± standard error.

Tamoxifen and flutamide, as reference chemicals in the antagonist assay, were tested first, and the results are shown in Figure 3A. As shown in the figure, tamoxifen inhibited the ERα transactivation which was elevated by spiked 25 pM 17β-estradiol. The concentration of tamoxifen used in the test was 10^−10^~10^−5^ M. The inhibitory activity was observed from a concentration of 10^−8^ M, and 50% inhibition was significant at 10^−7^ M. However, flutamide did not show any antagonistic effects in all the test concentrations. The results of the reference chemicals were very similar to those provided in the test guideline, confirming the validity of the performance criteria for the antagonist assay in this study.

BPP, BPS-MPE, and 2,4-BPS did not show the agonistic activity of ERα transactivation but showed antagonistic activity as shown in Figure 3B. The six chemicals (BPA, BPB, BPC, BPE, and BPZ) which showed agonistic effects on the ERα transactivation, did not inhibit ERα transcription activated by 25 pM 17β-estradiol in all the test concentrations (Figure 3C). IC_30_ and IC_50_ of the individual chemicals which showed ERα antagonistic effects were obtained from the concentration–response curve using the spreadsheet provided by the OECD TG 455 and are presented in Table 4.

### 3.4. ERα Transactivation of Binary Mixtures

#### 3.4.1. Mixtures of Agonist/Agonist

**Figure 4:**
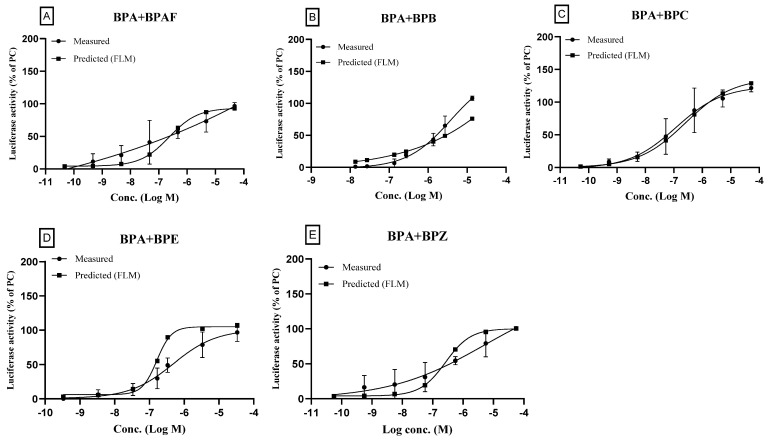
Effects of binary mixtures (Agonist + Agonist) on the estrogen receptor transcriptional activity: a comparison of the measured and the predicted values. For the test concentrations of binary mixtures, the highest concentration (master solution) was achieved by mixing the individual chemicals where the final concentration of each chemical was designed to be 100-fold EC_50_, respectively, for the full concentration–response curve. In case of BPB, the EC_50_ was too high to prepare 100-fold mixtures due to cytotoxicity at the master solution. So, the mixture containing BPB was designed to be 0.05-fold in binary mixture. The solution of the highest concentration was then serially diluted to measure the ERα transactivation. The measured results were shown with the predicted ones obtained by the full logistic model. The compositions of binary mixtures were as follows: (**A**): BPA + BPAF; (**B**): BPA + BPB; (**C**): BPA + BPC; (**D**): BPA + BPE; (**E**): BPA + BPZ. Three separate experiments were performed in the transactivation assay and data were represented as mean ± standard error.

The binary mixtures of BPA and other agonistic bisphenol compounds (BPAF, BPB, BPC, BPE, and BPZ), were prepared by mixing equi-effect concentrations as described in the methods section and agonistic effects of the binary mixtures were assayed. The results were compared to the predicted data obtained using FLM. The concentration–response curves of the binary mixtures (both the measured and the predicted values) are shown in Figure 4. As shown in the figures, the ERα transactivation increased as the mixture concentration increased both in the measured and predicted data. Furthermore, patterns of concentration–response curves of the measured and the predicted were very similar. The measured curves were very close to the predicted curves, respectively, which did not expect synergism or antagonism.

The RPC_Max_ (%), PC_10_, and PC_50_ were also very similar as shown in Table 5. Due to the same mode of action of the individual compounds, the mixture effect of binary bisphenol compounds seemed to be additive. When we calculated the model deviation ratio (MDR), most of the ratios were more than 0.5 and less than 2, which means that they were not in the range of synergism or antagonism.

#### 3.4.2. Mixtures of Antagonist/Antagonist

**Figure 5:**
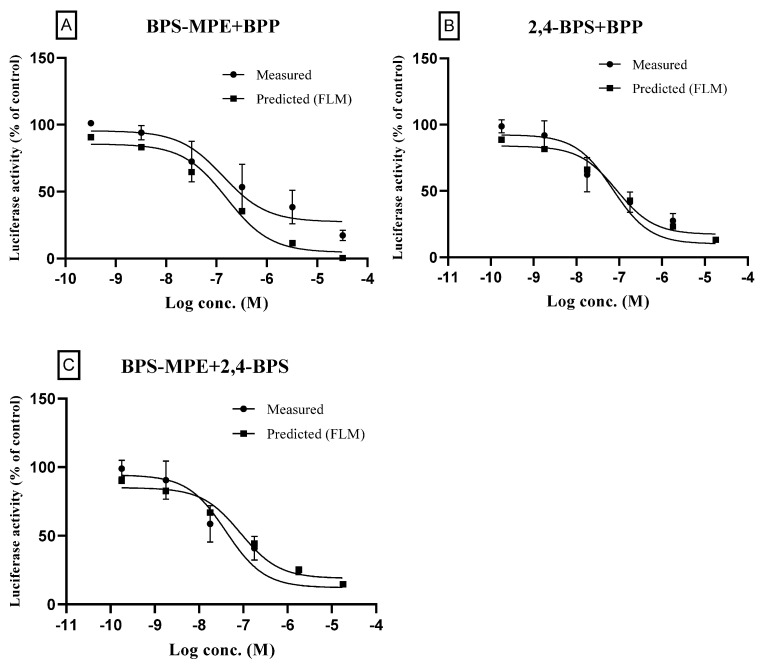
Effects of binary mixtures (Antagonist + Antagonist) on the estrogen receptor transcriptional activity: a comparison of the measured and the predicted values. For the test concentrations of binary mixtures, the highest concentration (master solution) was achieved by mixing the individual chemicals where the final concentration of each chemical was designed to be 100-fold IC_50_, respectively, for full concentration–response curve. The solution of the highest concentration was then diluted for the measurement of antagonistic activity. Test wells were spiked with 25 pM 17β-estradiol. The normalized mean values of the luminescence signal (Luciferase activity) of the test wells were divided by that of the spiked-in control (100%) to obtain the relative transcriptional activity of the binary mixture. The measured results were shown with the predicted ones obtained by the full logistic model. The compositions of binary mixtures were as follows: (**A**): BPS-MPE + BPP; (**B**): 2,4-BPS + BPP; (**C**): BPS-MPE + 2,4-BPS. Three separate experiments were performed in the transactivation assay and data were represented as mean ± standard error.

The binary mixtures were prepared by mixing two compounds with an equi-effect ratio of IC_50_ as described in the methods section. Three mixtures were prepared: BPS-MPE/BPP, 2,4-BPS/BPP, and BPS-MPE/2,4-BPS. The highest test concentration of the binary mixture was based on the IC_50_ of each compound, and the concentration was serially diluted. Test wells were spiked with 25 pM 17β-estradiol, and the normalized mean values of the luminescence signal (luciferase activity) of the test wells were divided by that of spiked control (100%). Concentration–response curves of binary mixtures of the antagonist compounds, both the measured and the predicted, are shown in Figure 5. As shown in the figure, the measured curves were very close to the predicted curves, respectively, which did not expect the synergism or antagonism. When the parameters of the concentration–response curves were calculated, they were also very similar as shown in Table 5. The MDRs, calculated as the ratio of the expected values and the measured values in the test concentrations, were within 0.50~2.00 indicating additive activity.

### 3.5. ERα Transactivation of Ternary Mixtures

**Figure 6:**
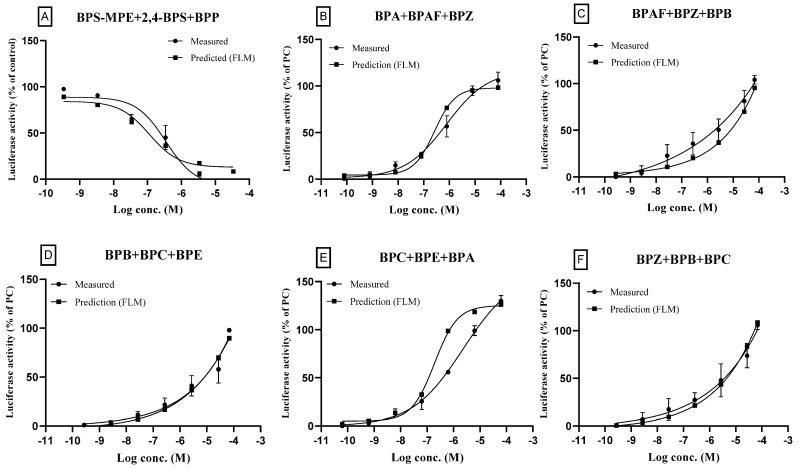
Effects of ternary mixtures on the estrogen receptor transcriptional activity: a comparison of the measured results and the predicted results. For the test concentrations of ternary mixtures, the highest test concentration was achieved by mixing the individual chemicals where the final concentration of each chemical was designed to be 100-fold EC_50_, respectively, for the full concentration–response curve. In the case of BPB, the EC_50_ was too high to prepare 100-fold mixtures due to cytotoxicity at the master solution. So, the mixture containing BPB was designed to be 0.25-fold in ternary mixture. The concentration of component chemicals in master solutions was designed for the full concentration–response curve. The solution of the highest test concentration was then diluted for the measurement and prediction of ERα transactivation. The components of ternary mixtures for antagonists (**A**) and agonists (**B**–**F**) are described in the figures. The measured results of concentration–response curves are shown with the predicted ones obtained by the full logistic model. Three separate experiments were performed in the transcription assay and data were represented as mean ± standard error.

A mixture of three antagonists (BPP, BPS-MPE, and 2,4-BPS) was prepared and tested for the antagonistic effects on ERα transactivation activated by 25 pM 17β-estradiol. As described in the methods section, the highest test concentration of the ternary mixture for antagonist assay was based on the sum of IC_50_ of respective individual compounds. The mixture of three antagonists (BPS-MPE, 2,4-BPS, and BPP) also showed an antagonistic effect as shown in Figure 6A. Five ternary mixtures prepared by combining six agonists (BPA, BPAF, BPB, BPC, BPE, and BPZ) were tested for the agonistic effect on ERα transactivation. The highest test concentration of the ternary mixture for agonist assay was based on the EC_50s_ of the respective individual three compounds. The results are shown in Figure 6B–F. Similarly to the binary mixtures, the concentration–response parameters of ternary mixtures also showed similarity between the measured and the predicted results, as shown in Table 6.

## 4. Discussion

Although the substitutes of BPA have been developed for safety or more useful products, these analogs also have adverse effects on the endocrine system. BPA and BPAF have been shown to activate the ERα and ERβ, while BPA (>100 nM) has a strong effect compared to BPAF (>1000 nM) [39]. On the ER transcriptional assay performed by Liu et al. in another study, BPAF, BPB, BPC, BPE, and BPZ were reported to function as full activators or agonists for ERα, but they completely inhibited the ERβ [40,41].

In our previous study, BPA, BPF, and BPS showed strong ERα transcriptional activity [29]. BPB has been demonstrated to be anti-androgenic as well as estrogenic and was also found to exert higher estrogenic activity than BPA [42,43]. In this study, as shown in Table 4, the RPC_Max_ of the BPC was higher and PC_50_ was lower than those of BPA, which supports the strong estrogenic activity of BPC. The estrogenic activity was in this order, BPC > BPE > BPZ > BPB > BPAF > BPA when they were compared based on the RPC_Max_. It suggested that the BPA substitutes may also have strong endocrine-disrupting effects. Other BPA analogs including BPP, BPS-MPE, and 2,4-BPS did not show agonistic effects on the ERα transactivation but showed antagonistic effects (Figure 2D and Figure 3).

Compared to BPS, only a few studies on 2,4-BPS and BPS-MPE have been reported. BPS is known to have strong agonistic activity in ERα transactivation, and the RPC_Max_ of ERα transactivation was shown to be 72% [32]. However, BPS-MPE and 2,4-BPS, which are the structural analogs of BPS, did not show agonistic activity but showed antagonistic activity in this study. The antagonistic activity of 2,4-BPS was stronger than that of BPS-MPE, compared based on LogIC_50,_ as shown in Table 4. Further investigations on the differences in the effects of BPS and 2,4-BPS are necessary because the two compounds are structurally similar and are only different in the position of the hydroxyl group. BPP also has antagonistic activity. Only a few reports for the BPP are available on the endocrine-disrupting effects [44].

Recently, an association study on the combined exposures to bisphenol compounds and endocrine disruption has been reported, where the concentration of BPA, BPF, and BPS in the urine of pregnant women was analyzed and the birth outcome was investigated [45]. Furthermore, it has been reported that the hazard index for the deterioration of semen quality might be increased by the combined exposure to a mixture of BPA, BPF, and BPS [46]. A study on the association of bisphenol compounds with odds of polycystic ovarian syndrome (PCOS) in women of childbearing age, showed that mixed exposure to seven bisphenol compounds (BPA, BPAP, BPAF, BPB, BPS, BPP, and BPZ) was found to be positively associated with the odds of PCOS (odds ratio = 1.26), while the odds ratio of single bisphenol compounds was less than 1 [47]. Although the independent action of bisphenol compound has been the main principle in mixture toxicity, co-exposure to these chemicals may induce hazardous health effects potentially associated with a complex body burden of different origins [25].

In our study, binary or ternary mixtures of bisphenol A and its structural analogs were evaluated for the interaction of single compounds on the ERα transactivation. When the mixtures were prepared and tested, they showed additive activity in both the agonists/agonist and antagonists/antagonist preparations in the test concentrations (Figure 4, Figure 5 and Figure 6). There were no significant differences between the measured values and the predicted values. The MDR values were more than 0.5 and less than 2, which indicated the additive activity. Many chemicals with estrogenic activity could be contained in consumer products and may be exposed to humans simultaneously. Therefore, the mixture toxicity of estrogenic chemicals in consumer products needs to be assessed. However, the combinations of chemicals, the number of compounds, and the concentration of individual components in the mixture are too numerous to test efficiently. According to our study, we found that the measured ERα transcription activity results closely matched the calculated results obtained from FLM. This suggests that it may not be necessary to conduct a separate test for each combined mixture. Understanding the parameters of concentration–response curves for each chemical component, however, allows one to determine the transcription activity of multiple mixtures. Additionally, the in silico model used in this study may provide a good tool for the prediction of an in vitro transcription assay.

## 5. Conclusions

Bisphenol A and its eight substitutes, BPAF, BPB, BPC, BPE, BPP, BPS-MPE, BPZ, and 2,4-BPS, which are used in the manufacture of industrial and consumer products were selected and evaluated for the transactivation of estrogen receptor alpha (ER α). The test methods were based on the OECD TG 455. Among the chemicals, six compounds were found to be agonistic, and three compounds were antagonistic to the ERα transcription activity. Binary and ternary mixtures including different combinations of agonist/agonist and antagonist/antagonist were prepared on an equi-effect concentration basis and tested for ERα transcription activity. The activity of the mixtures showed concentration-dependent responses. The concentration–response curves were also predicted by using FLM. The measured and the predicted response curves showed close similarities. Overall results suggest that the effect of bisphenol compound is additive in binary and ternary mixtures.

## Figures and Tables

**Table 1:** Bisphenol A and its structural analogues for estrogen receptor transactivation assay.

No.	Chemical Name	CAS No.	M.W.	Test Con. (M)	Vendors	Structure
1	4,4′-Isopropylidenediphenol (BPA)	80-05-7	228.29	10^−11^–10^−5^	A	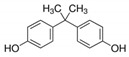
2	4,4′-(Hexafluoroisopropylidene)diphenol (BPAF)	1478-61-1	336.23	10^−11^–10^−5^	A	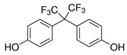
3	2,2-Bis(4-hydroxyphenyl)butane (BPB)	77-40-7	242.32	10^−11^–10^−5^	B	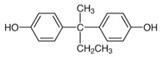
4	2,2-Bis(4-hydroxy-3-methylphenyl)propane (BPC)	79-97-0	256.35	10^−11^–10^−5^	B	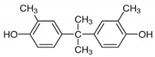
5	4,4′-Ethylidenediphenol (BPE)	2081-08-05	214.26	10^−11^–10^−5^	B	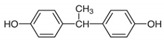
6	α,α′-Bis(4-hydroxyphenyl)-1,4-diisopropylbenzene (BPP)	2167-51-3	346.46	10^−11^–10^−6^	B	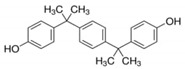
7	4-Benzyloxyphenyl 4-Hydroxyphenyl Sulfone (BPS-MPE)	63134-33-8	340.39	10^−11^–10^−6^	B	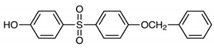
8	4,4′-Cyclohexylidenebisphenol (BPZ)	843-55-0	268.35	10^−11^–10^−5^	A	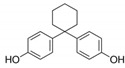
9	2,4′-Dihydroxydiphenyl Sulfone (2,4-BPS)	5397-34-2	250.27	10^−11^–10^−6^	B	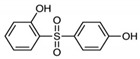

A: Sigma-Aldrich; B: Tokyo Chemical Industry.

**Table 2:** Chemicals for mixture preparations and their parameters obtained from concentration–response curves of agonistic activity or antagonistic activity.

Chemicals		EC_50_		IC_50_		MIN	MAX	Hill Slope
		(M)	(Log M)	(M)	(Log M)			
Agonist	BPA	2.31 × 10^−7^	−6.64	-	-	2.66	95.14	0.66
	BPAF	2.40 × 10^−7^	−6.62	-	-	5.00	93.34	1.11
	BPB	2.72 × 10^−4^	−3.57	-	-	−6.41	424.1	0.32
	BPC	2.91 × 10^−7^	−6.54	-	-	−3.95	180.3	0.45
	BPE	1.04 × 10^−7^	−6.98	-	-	2.12	120.6	7.36
	BPZ	3.26 × 10^−7^	−6.49	-	-	4.04	108.5	1.52
Antagonist	BPP	-	-	1.62 × 10^−7^	−6.79	−5.64	92.12	−0.56
	BPS-MPE	-	-	1.63 × 10^−7^	−6.79	−4.52	96.26	−0.51
	2,4-BPS	-	-	1.50 × 10^−8^	−7.83	19.75	93.86	−0.40

The parameters were calculated using GraphPad Prism. EC: effective concentration; MIN: minimum values of the curves; MAX: maximum values of the curves.

**Table 3:** Concentrations and ratios of component chemicals in the master solution of mixtures.

Chemicals			Ind. Conc. (M)	Total Conc.		Ratio
				(M)	(Log M)	
Binary	Ago + Ago	BPA + BPAF	2.3 × 10^−5^ + 2.4 × 10^−5^	4.7 × 10^−5^	−4.33	1.0:1.0
		BPA + BPB ^(1)^	1.2 × 10^−8^ +1.4 × 10^−5^	1.4 × 10^−5^	−4.87	1.0:1174.6
		BPA + BPC	2.3 × 10^−5^ + 2.9 × 10^−5^	5.2 × 10^−5^	−4.28	1.0:1.3.0
		BPA + BPE	2.3 × 10^−5^ + 1.1 × 10^−5^	3.4 × 10^−5^	−4.47	1.0:2.2
		BPA + BPZ	2.3 × 10^−5^ + 3.3 × 10^−5^	5.6 × 10^−5^	−4.25	1.0:1.4
	Anta + Anta	BPS-MPE + BPP	1.6 × 10^−5^ + 1.6 × 10^−5^	3.3 × 10^−5^	−4.49	1.0:1.0
		2,4-BPS + BPP	1.5 × 10^−6^ + 1.6 × 10^−5^	1.8 × 10^−5^	−4.75	1.0:10.8
		2,4-BPS + BPS-MPE	1.5 × 10^−6^ + 1.6 × 10^−5^	1.8 × 10^−5^	−4.75	1.0:10.9
Ternary	Ago + Ago + Ago	BPA + BPAF + BPZ	2.3 × 10^−5^ + 2.4 × 10^−5^ + 3.3 × 10^−5^	8.0 × 10^−5^	−4.10	1.0:1.0:1.4
		BPAF + BPZ + BPB ^(2)^	6.0 × 10^−8^ + 8.1 × 10^−8^ + 6.8 × 10^−5^	6.8 × 10^−5^	−4.17	1.0:1.4:1131.6
		BPE + BPC + BPB ^(2)^	2.6 × 10^−8^ + 7.3 × 10^−8^ + 6.8 × 10^−5^	6.8 × 10^−5^	−4.17	1.0:2.8:2611.0
		BPE + BPA + BPC	1.0 × 10^−5^ + 2.3 × 10^−5^ + 2.9 × 10^−5^	6.3 × 10^−5^	−4.20	1.0:2.2:2.8
		BPC + BPZ + BPB ^(2)^	7.3 × 10^−8^ + 8.1 × 10^−8^ + 6.8 × 10^−5^	6.8 × 10^−5^	−4.17	1.0:1.1:935.6
	Anta + Anta + Anta	2,4-BPS + BPP + BPS-MPE	1.5 × 10^−6^ + 1.6 × 10^−5^ + 1.6 × 10^−5^	3.4 × 10^−5^	−4.47	1.0:10.8:10.9

The concentration of component chemicals in master solutions are 100-fold EC_50_ (agonists) or 100-fold IC50 (antagonists) except the mixture ^(1)^ and ^(2)^. The master solution was diluted serially for the assay as designated in the figures. ^(1)^: The concentration of component chemicals in master solutions are 0.05 × EC_50_ (agonists). ^(2)^: The concentration of component chemicals in master solutions are 0.25 × EC50 (agonists).

**Table 4:** Parameters obtained from concentration–response curves of agonists or antagonists for the estrogen receptor transactivation.

Chemicals		RPC_Max_ (%)	PC_10_	PC_50_	IC_30_	IC_50_
			(Log M)	(Log M)	(Log M)	(Log M)
Agonist	BPA	88.35	−8.36	−6.63	-	-
	BPAF	91.34	−8.35	−6.57	-	-
	BPB	104.53	−8.16	−6.00	-	-
	BPC	148.20	−9.24	−7.33	-	-
	BPE	138.06	−8.42	−7.07	-	-
	BPZ	107.87	−7.87	−6.58	-	-
Antagonist	BPP	-	-	-	−7.39	−6.68
	BPS-MPE	-	-	-	−7.57	−6.74
	2,4-BPS	-	-	-	−8.84	−8.00

PC: positive concentration; IC: inhibition concentration; RPC_Max_: maximum level of response induced by a test chemical, expressed as a percentage of the response induced by 1 nM 17β-estradiol (positive chemical) on the same plate. The parameters were obtained using the spreadsheet provided by the OECD Test guideline.

**Table 5:** Parameters obtained from concentration–response curves of binary mixtures for the estrogen receptor transactivation.

Chemicals		RPC_Max_ (%)		PC_10_ (Log M)		PC_50_ (Log M)		IC_30_ (Log M)		IC_50_ (Log M)	
		Meas	Pred	Meas	Pred	Meas	Pred	Meas	Pred	Meas	Pred
Ago + Ago	BPA + BPAF	96.7	92.8	−9.43	−8.16	−6.74	−6.63	-	-	-	-
	BPA + BPB	134.6	146.0	−6.79	<−7.56	−5.78	−5.54	-	-	-	-
	BPA + BPC	121.6	128.7	−9.12	−8.84	−7.22	−7.06	-	-	-	-
	BPA + BPE	96.8	106.5	−7.97	−8.00	−6.44	−6.86	-	-	-	-
	BPA + BPZ	100.8	100.4	−9.72	−8.00	−6.44	−6.65	-	-	-	-
Anta + Anta	BPS-MPE + BPP	101.2	90.7	-	-	-	-	−7.35	−7.77	−6.26	−6.98
	2,4-BPS + BPP	98.8	88.7	-	-	-	-	−8.01	−8.00	−7.16	−7.06
	BPS-MPE + 2,4-BPS	99.0	90.2	-	-	-	-	−8.10	−7.94	−7.26	−7.00

Ago: agonist; Anta: antagonist; Meas: measured values; Pred: predicted values; PC: positive concentration; IC: inhibition concentration; RPC_Max_: maximum level of response. The parameters were obtained using the spreadsheet provided by the OECD TG 455.

**Table 6:** Parameters obtained from concentration–response curves of ternary mixtures for the estrogen receptor transactivation.

Chemicals	RPC_Max_ (%)		PC_10_ (Log M)		PC_50_ (Log M)		IC_30_ (Log M)		IC_50_ (Log M)	
	Meas	Pred	Meas	Pred	Meas	Pred	Meas	Pred	Meas	Pred
BPA + BPAF + BPZ	106.1	98.2	−8.50	−7.95	−6.33	−6.61	-	-	-	-
BPAF + BPZ + BPB	104.1	95.4	−8.00	−7.73	−5.60	−5.17	-	-	-	-
BPB + BPC + BPE	97.9	89.7	−8.00	−7.23	−5.03	−5.15	-	-	-	-
BPC + BPE + BPA	129.8	127.0	−8.53	−8.61	−6.40	−6.94	-	-	-	-
BPZ + BPB + BPC	105.8	108.7	−8.26	−7.51	−5.48	−5.40	-	-	-	-
BPS-MPE + 2,4-BPS + BPP	97.6	89.1	-	-	-	-	−7.65	−7.91	−6.71	−7.00

Meas: measured values; Pred: predicted values; PC: positive concentration; IC: inhibition concentration; RPC_Max_: maximum level of response. The parameters were obtained using the spread sheet provided by the OECD Test guideline.

## Data Availability

The data presented in this study are available on request from the corresponding author.
